# CGRP-targeted medication in chronic migraine - systematic review

**DOI:** 10.1186/s10194-024-01753-y

**Published:** 2024-04-05

**Authors:** Renato Oliveira, Raquel Gil-Gouveia, Francesca Puledda

**Affiliations:** 1 Headache Group, Wolfson SPaRRC, Institute of Psychiatry, Psychology and Neuroscience, King’s College London, London, SE5 9PJ UK; 2 Neurology Department, Barking, Havering and Redbridge University Hospitals NHS Foundation Trust, London, UK; 3 Hospital da Luz Headache Center, Neurology Department, Hospital da Luz Lisboa, Lisbon, Portugal; 4 Center for Interdisciplinary Research in Health, Universidade Católica Portuguesa, Lisbon, Portugal

**Keywords:** Migraine, Chronic migraine, CGRP, anti-CGRP, Medication overuse

## Abstract

**Background:**

Chronic migraine is a highly debilitating condition that is often difficult to manage, particularly in the presence of medication overuse headache. Drugs targeting the calcitonin gene-related peptide (CGRP), or its receptor have shown promising results in treating this disorder.

**Methods:**

We searched Pubmed and Embase to identify randomized clinical trials and real-world studies reporting on the use of medication targeting the calcitonin gene-related peptide in patients with chronic migraine.

**Results:**

A total of 270 records were identified. Nineteen studies qualified for the qualitative analysis. Most studies reported on monoclonal antibodies targeting CGRP (anti-CGRP mAbs), that overall prove to be effective in decreasing monthly migraine days by half in about 27.6–61.4% of the patients. Conversion from chronic to episodic migraine was seen in 40.88% of the cases, and 29–88% of the patients stopped medication overuse. Obesity seems to be the main negative predictor of response to anti-CGRP mAbs. There is no evidence to suggest the superiority of one anti-CGRP mAb. Despite the lack of strong evidence, the combination of anti-CGRP medication with onabotulinumtoxinA in chronic migraine is likely to bring benefits for resistant cases. Atogepant is the first gepant to demonstrate a significant decrease in monthly migraine days compared to placebo in a recent trial. Further, anti-CGRP mAb and gepants have a good safety profile.

**Conclusion:**

There is strong evidence from randomized trials and real-world data to suggest that drugs targeting CGRP are a safe and effective treatment for chronic migraine.

**Supplementary Information:**

The online version contains supplementary material available at 10.1186/s10194-024-01753-y.

## Background

Chronic migraine (CM) is a disabling primary headache that affects 3–4% of people with migraine [1]. CM is the most prevalent type of headache in a tertiary headache center [2]. The management of CM is challenging due to several associated factors such as medication overuse, which affects 1 to 2% of the general population and about 11–70% of people with CM, as well as superimposed comorbid conditions [3–5]. For these reasons, CM causes a wide range of personal, familial, and economic societal burdens [6, 7] Although patients with CM should receive preventive medication according to existing guidelines on migraine [8], until 2018 the therapeutic arsenal was mostly limited to two evidence-based treatments – topiramate and subcutaneous onabotulinumtoxin-A (BTX-A), and other prophylactic drugs tested mostly in episodic migraine [9]. Non-adherence to oral medication due to poor tolerance is one of the driving factors leading to treatment failure in CM, with only 17–20% of adherence after 1 year [10, 11]. Up to 30–50% of CM cases do not respond to BTX-A [12]. Additionally, while people with CM often benefit from BTX-A, they may continue to experience migraine attacks at a frequency that meets the criteria for receiving additional preventive treatments [13].

Following the introduction of monoclonal antibodies targeting calcitonin gene-related peptide (CGRP) or its receptor (anti-CGRP mAbs), there are currently four anti-CGRP mAbs with evidence in both episodic and CM [14, 15]. There are also three oral small molecule CGRP receptor antagonists that belong to the gepants drug class. Of the three second-generation gepants, both atogepant and rimegepant are FDA and EMA approved for migraine prophylaxis, and rimegepant is also approved by NICE. However, only atogepant is currently approved for prevention treatment in CM, and only in the USA. These new classes of drugs changed the paradigm of migraine treatment. However, there are still unmet needs for a significant number of patients with migraine, particularly those qualifying as resistant or refractory [16]. There are also ongoing questions regarding appropriate treatment duration with the novel drugs, the predictive factors of response, as well as the potential benefit of combination with other preventives and switching among classes.

In this review, we aimed to explore the available data on efficacy, safety, and other selected topics such as predictive factors of response of CGRP targeted medication in CM, and potential impact on medication overuse headache (MOH). We also looked into the possible interaction between these new drug class with BTX-A.

## Methods

We performed a systematic review, following the PRISMA (Preferred Reporting Items for Systematic Reviews and Meta-Analyses) guidelines [17]. The data that support the findings of this study are available from the corresponding author upon reasonable request.

### Eligibility criteria

We considered both phase III and IV clinical trials (RCT) and real-world studies (RWS) reporting on patients with CM that received at least one CGRP-targeted medication, including both anti-CGRP mAbs and gepants. We included studies focusing on their efficacy, safety, and several other aspects such as responsive predictive factors, effect of discontinuation, and combination with other available treatments such as BTX-A. We excluded single case reports, case series describing fewer than 40 patients with CM, narrative reviews, and reports with very short follow-up (< 1 month after medication). We also excluded studies on episodic migraine or non-migraine headaches. However, we considered studies including both episodic migraine (EM) and CM. We considered articles in English, Spanish, French, Italian, German, and Portuguese.

### Search strategy

A systematic search using combinations of keywords was performed in MEDLINE/Pubmed database, on the 30th of June 2023. A second database, Embase, was used to search for additional potential studies. The search strategy combined the main terms “headache, migraine, chronic migraine, CGRP, anti-CGRP, erenumab, galcanezumab, fremanezumab, eptinezumab, gepants, rimegepant or atogepant” (details in supplementary data). Potential eligible studies and selected study reference lists were crosschecked for additional studies. Additional data from international conference abstracts, clinical trial websites, and proceedings were analyzed for unpublished data. Identified studies were screened for potential eligibility by title and abstract analysis. The full text of potentially eligible studies was then screened to meet the inclusion criteria and exclusion criteria.

### Assessment of study quality

The risk of bias for each eligible study was assessed with the Newcastle-Ottawa Quality Assessment Scale for cohort studies tool using efficacy as an intervention (treatment with anti-CGRP) outcome (supplementary data).

## Results

### Study selection

Our initial search retrieved 270 records, of which forty-nine were retained for full-text analysis. The majority (*n* = 44) were studies on anti-CGRP mAbs, while a small number of papers focused on gepants (*n* = 5). Finally, *n* = 19 studies were selected for qualitative analysis. Of this selection, ten studies were included for reviewing the efficacy of the anti-CGRP mAbs in CM, which included five phase III RCT [18–22] and five RWS [23–27]. The other included studies had information on MOH [28–30], predictive factors [31], discontinuation of anti-CGRP mAbs treatment [32], and potential interaction between anti-CGRP mAbs and BTX-A [33–35]. The results on gepants in CM were based on the results of one clinical trial [36]. Of the 19 studies, ten studies had a moderate risk of bias (supplementary data).

### Overall efficacy of anti-CGRP mAbs in chronic migraine

The efficacy of anti-CGRP mAbs was mostly assessed by considering changes in headache frequency. In most studies, the primary endpoint was the change from baseline of the average number of monthly headache days (MHDs) or monthly migraine days (MMDs), measured as the least-squares mean during a pre-determined follow-up period. Common secondary outcomes included: the proportion of patients with ≥ 50% and ≥ 75% reduction in the average number of MHDs and/or MMDs, the average number of days with use of any acute headache medication per month, the conversion rate from CM to EM, the conversion rate from MOH to non-MOH, and the impact on headache-related disability (Table 1).

**Table 1:** Available information on different outcomes among selected studies on anti-CGRP monoclonal antibodies in chronic migraine

Study	Reduction in MHDs			Reduction in MMDs			Reduction on HIT-6			Reduction on monthly pain medication intake		
Follow-up (months)	3	6	12	3	6	12	3	6	12	3	6	12
[32]				X	X	X				X	X	X
[23]	X	X					X			X	X	
[24]												
[25]	X	X					X	X				
[26]	X	X		X	X		X	X		X	X	
[21]				X		X						
[18]	X			X			X					
[19]				X								
[22]				X			X			X		
[20]	X		X	X		X						

HIT-6 – Headache Impact Test 6; MHDs - monthly headache days; MMDs - monthly migraine days

The baseline characteristics of the cohorts included in both RCT and RWS studies were comparable, with similar mean age, sex ratio, MMD, and MHD, while there was considerable heterogeneity between RCT and RWS in the prevalence of MOH (0-63.6% vs. 54–100%, respectively) and concomitant prophylactic medication (14.6–44.7% vs. 57–59%, respectively) (Tables 2 and 3). Additionally, in 4 of the 5 RCT participants with continuous headache (with no headache-free period), and/or daily headache were excluded [18–21]. The studies included different anti-CGRP mAbs: erenumab [21, 23, 25, 26], fremanuzumab [19, 22], galcanezumab [20], eptinezumab [18], and 2 studies included patients receiving either erenumab, fremanezumab or galcanezumab [24, 27].

**Table 2:** Characteristics of phase III randomized clinical trials on anti-CGRP monoclonal antibodies in chronic migraine

Study	Country	Sample	Key inclusion criteria	Selective exclusion criteria	MOH, %	Follow-up, months	Key results	Safety – Any adverse events
[18]	International, multicentric	1072: Eptinezumab 100 mg (356) Eptinezumab 300 mg (350) Placebo (366)	18–65 yo 15–26 headache days per month	Continuous daily headache, Opioids > 4days/month Comorbid pain disorder	40.2	3	**Mean reduction in MMD from baseline** 16.1 to 8.5 days (100 mg) vs. 16.1 to 7.9 days (300 mg) vs. 16.2 to 10.5 days (P) **≥ 50% reduction in headache frequency**: 57.6% (100 mg) vs. 61.4% (300 mg) vs. 39.3% (P) **Mean change in HIT-6 from baseline**: -6.2 (100 mg) vs. -7.3 (300 mg) vs. -4.5 points (P)	43.5% (100 mg) vs. 52% (300 mg) vs. 46.7% (P)
[19]	Multicentric, Japan and Korea	569: Fremanezumab-monthly (187) Fremanezumab-quarterly (189) Placebo (191)	18–70 yo	Unremiting headache (less than 4 days of headache free/month) Failure of ≥ 2 preventives	0	3	**Mean reduction in MMD from baseline** 16.4 to 11.5 days (monthly) vs. 15.2 to 11.1 days (quarterly) vs. 15.4 to 12.6 days (P) **≥ 50% reduction in headache frequency**: 29% (monthly) vs. 29.1% (quarterly) vs. 13.2% days (P) **Mean change in HIT-6 from baseline**: -8.1 (monthly) vs. -8 (quarterly) vs. -6.5 points (P)	61.7% (monthly) vs. 61.1% (quarterly) vs. 61.8% (P)
[20]	International, multicentric	1113: Galcanezumab 120 mg (273) Galcanezumab (274) Placebo (558)	18–65 yo	Persistent daily headache Failure of ≥ 3 preventives Cluster headache Stroke history Opioids > 3 days/month	63.6	3 plus Open label extension of 9 months	**Mean reduction in MMD from baseline** 19.4 to 14.6 (120 mg) vs. 19.2 to 14.6 (240 mg) vs. 19.6 to 16.9 days (P) **≥ 50% reduction in headache frequency**: 27.6% (120 mg) vs. 27.5% (240 mg) vs. 15.4% (P) **Mean change in MIDAS from baseline**: -20.3 (120 mg) vs. -17 (240 mg) vs. -11.5 points (P)	57% (120 mg) vs. 58% (240 mg) vs. 50% (P)
[22]	International, multicentric	1130: Fremanezumab-monthly (376) Fremanezumab-quarterly (379) Placebo (375)	18–70 yo	Failure of ≥ 2 preventives Opioids/Barbiturates > 4days/month	Not mentioned	3	**Mean reduction in MMD from baseline** 16.4 to 11.5 days (monthly) vs. 15.2 to 11.1 days (quarterly) vs. 15.4 to 12.6 days (P) **≥ 50% reduction in headache frequency**: 41% (monthly) vs. 38% (quarterly) vs. 18% days (P) **Mean change in HIT-6 from baseline**: -6.8 (monthly) vs. -6.4 (quarterly) vs. -4.5 points (P)	70% (monthly) vs. 70% (quarterly) vs. 64% (P)
[21]	International, multicentric	656: Erenumab 70 mg (191) Erenumab 140 mg (190) Placebo (286)	18–65 yo	Continuous daily headache Failure of ≥ 3 preventives Fibromyalgia cluster headache, hemiplegic migraine	41.8	3 plus Open label extension of 9 months	**Mean reduction in MMD from baseline** 17.9 to 11.3 days (70 mg) vs. 17.8 to 11.2 days (140 mg) vs. 18.2 to 14 days (P) **≥ 50% reduction in headache frequency**: 40% (70 mg) vs. 41% (140 mg) vs. 2.3% (P) **Mean change in MIDAS from baseline**: -20.3 (120 mg) vs. -17 (240 mg) vs. -11.5 points (P)	44% (70 mg) vs. 47% (140 mg) vs. 39% (P)

CM – chronic migraine; EM – episodic migraine; HIT-6 – Headache Impact Test 6; MHDs - monthly headache days; MMDs - monthly migraine day; MOH – medication overuse headache; P – Placebo

**Table 3:** Characteristics of real word-studies on anti-CGRP monoclonal antibodies in chronic migraine

Study	Type of study Country	Sample	Inclusion criteria	MOH, %	Anti-CGRP mAb	Follow-up, months	Outcomes	Safety – Adverse events
[32]	Monocentric Prospective Italy	203	Chronic migraine Failure of ≥ 3 preventive treatments	84.8	Erenumab (47.2%) Galcanezumab (36.5%) Fremanezumab (16.3%)	12	**Mean reduction in MMD from baseline** 8.4 to 13.2 **≥ 50% reduction in headache frequency**: 36.4–56.8%	7.9%
[30]	Multicentric Prospetive Italy	149	Chronic migraine with MOH Failure of ≥ 3 preventive treatments plus failure of BTX-A	100	Erenumab 70 ◊ 140 mg	3	**Reduction in MHDs from** 25.4 ± 5.4 to 14.1 ± 8.6 **≥ 50% reduction in headache frequency** 51% **Percentage of cases converted from CM to EM**: 64% **Mean change in HIT-6 from baseline**: -9.5 points	32%
[24]	Monocentric Prospective Italy	303	Chronic migraine with MOH	100	Erenumab (48.6%) Galcanezumab (20.6) Fremanezumab (30.6%)	3–12	**≥ 50% reduction in headache frequency** 80% **Percentage of cases converted from CM to EM**: 88% **Change in MIDAS**: 56.5 to 13.1	19%
[26]	Monocentric Prospective United Kingdom	164	Chronic migraine Failure of ≥ 3 preventive treatments	54	Erenumab	6	**Mean reduction in MMD from baseline** 7.5 days **≥ 50% reduction in headache frequency** 49% **Percentage of cases converted from CM to EM**: 40% **Mean change in HIT-6 from baseline**: -7.5 points	48%
[25]	Monocentric Prospective Italy	70	Chronic migraine Failure of ≥ 4 preventive treatments	91.4	Erenumab 70 ◊ 140 mg	6	**Reduction in MMDs from** 21.1 +/- 0.7 to 11.4 +/- 0.9 days **≥ 50% reduction in headache frequency** 53% **Percentage of cases converted from CM to EM**: 66% **Mean change in HIT-6 from baseline**: -6.4 points	25.7%

**Fig. 1:**
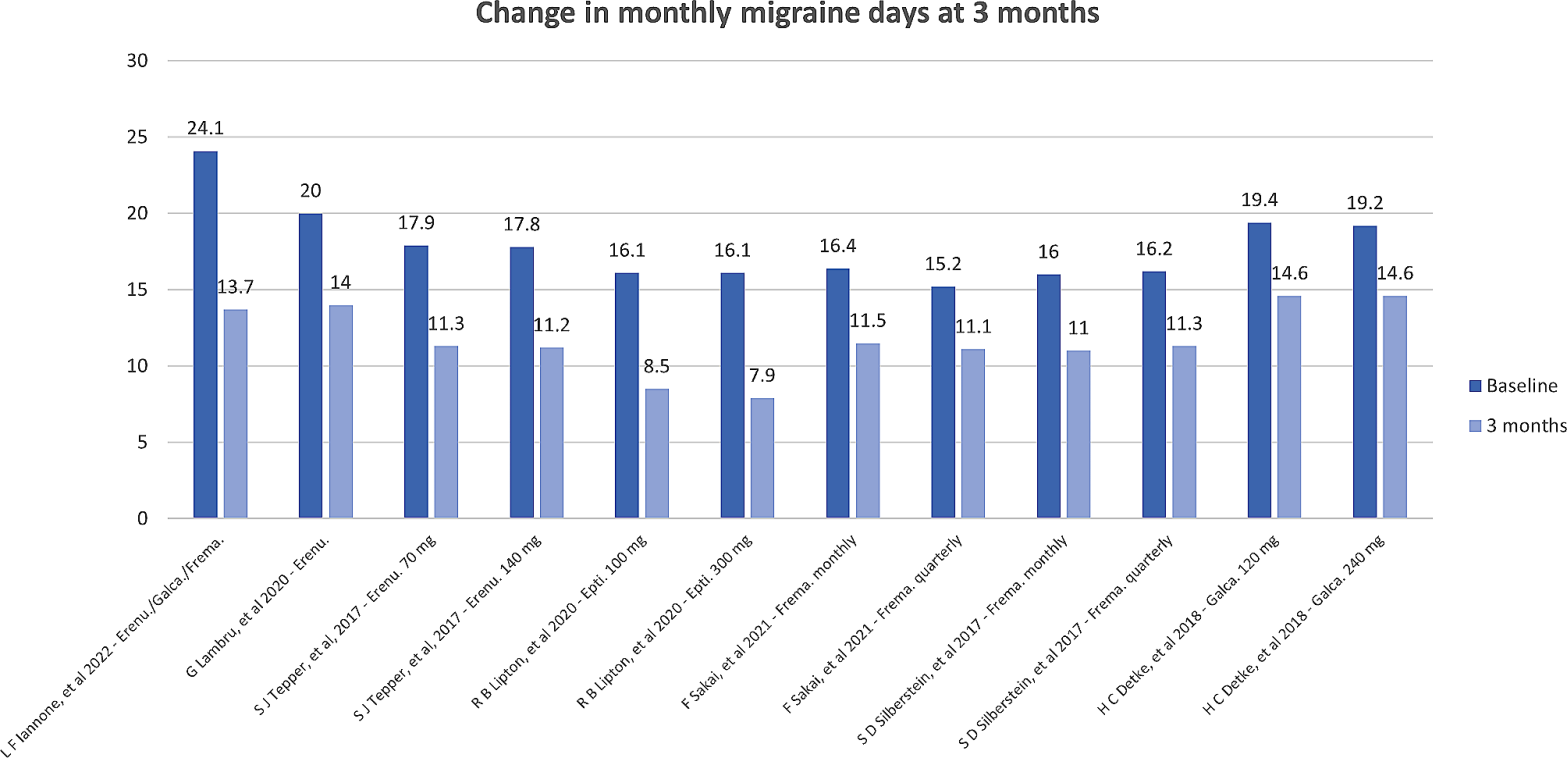
Change in monthly migraine days after 3 months of treatment with anti-CGRP mAb. Legend: Epti: eptinezumab; Erenu: erenumab; Frema: fremanezumab; Galca: galcanezumab

**Fig. 2:**
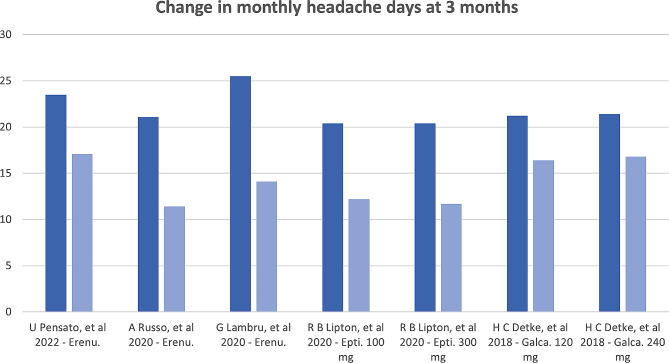
Change in monthly headache days after 3 months of treatment with anti-CGRP mAb. Legend: Epti: eptinezumab; Erenu: erenumab; Frema: fremanezumab; Galca: galcanezumab

Follow-up to assess efficacy ranged from 3 to 12 months, with most available data present at the 3-month evaluation. In all studies, the anti-CGRP mAbs were effective in significantly decreasing the headache frequency, with similar results between RCT and RWS (Figs. 1 and 2). The proportion of patients with ≥ 50% reduction in MMDs ranged from 27.6 to 61.4% [20, 21, 27], and in MHDs from 29 to 80% [19, 22–26]. The highest response (80%) was seen in the Curone et al. cohort [24], which included 303 patients with CM and MOH. This study, however, only included participants who were not taking any other preventive, and 32% had never received a preventive treatment before, which might have skewed the response to a more positive effect. In the other RWS cohorts, CM patients were included if they had failed at least 3 preventive drugs. There were also high prevalences of MOH patients, making these studies more representative of difficult-to-treat migraine populations [23, 25, 26].

The benefit from anti-CGRP mAbs on CM was seen as early as the first dose. In their cohort of *n* = 70 patients, Russo et al. found that 60% had a ≥ 30% reduction in headache days in the month after the first erenumab injection [25] A single dose of erenumab converted 27% of patients to EM in another study [26]. At 6 months the rate of conversion from CM to EM in the RWS ranged from 40 to 70% [25, 26]. The rapid effect of the anti-CGRP mAbs appears to be particularly relevant for eptinezumab, which had a > 50% reduction in migraine prevalence on the day after dosing [18].

All studies reported good results regarding secondary outcomes. In the RCT the reduction in analgesic consumption was consistently and significantly higher in the treated group than in the placebo group [19–22]. A ≥ 50% reduction in monthly pain medication intake was seen in 70% in one study at 6 months follow-up in one study [25]. A significant reduction in headache disability measured by HIT-6 was found in both RWS [23, 25, 26] and RCT [18, 19, 22]. Iannone et al. assessed the impact of one year of treatment with three mAbs (erenumab – 47.2%, galcanezumab-36.5%, and fremanezumab-16.3%) on MIDAS scores among *n* = 203 treatment-resistant CM patient [27]. Results showed a 50% reduction in 89.5% of the participants at 6 months, and in 100% at 12 months, a much higher number than when considering change in MMDs for efficacy assessment [27].

The antibodies also had a positive effect on headache-associated symptoms such as premonitory symptoms and allodynia [25]. However, evidence is still lacking on the effect of anti-CGRP medication in non-headache phases of migraine.

Regarding common comorbidities associated with CM, selective studies found a significant improvement in mood, anxiety, and sleep after treatment with anti-CGRP mAbs [25]. In one study, however, erenumab did not result in a significant reduction in the cognitive symptoms associated with migraine attacks (assessed by the subjective COGnitive impairments scale), either at the third or sixth month of treatment [25].

### Predictive factors of anti-CGRP mAbs response

Some studies have found that responsiveness to anti-CGRP mAbs could be related to different demographic and clinical features. One multicenter RWS involved 20 headache centers and 864 patients treated with anti-CGRP mAbs for at least 6 months and looked into possible predictors of response [31]. Among people with CM (75.9% of the population) the response to anti-CGRP mAbs was positively associated with unilateral autonomic signs, unilateral pain plus autonomic signs, and unilateral pain plus allodynia, whereas it was negatively associated with obesity [31]. The authors did not find any significant results with triptan response, BTX-A response, prior treatment failures, and disability HIT-6 score [31]. Regarding headache frequency, most studies show that a lower clinical burden at baseline is associated with a better response to anti-CGRP mAbs [27]. Russo et al. also found that disease duration is negatively associated with response to anti-CGRP mAbs [25].

### Medication overuse headache

Real-life data have consistently shown that anti-CGRP mAbs are effective in the treatment of CM with MOH and that there are no differences in response between CM patients with or without MOH [26]. The conversion from medication overuse to non-medication overuse was seen in up to 57–64% of people with CM [23, 25]. Data from a subgroup analysis of one RCT [28] showed better responses to erenumab in both the medication overuse and non-medication overuse subgroups than in the placebo group. Most people who switched to non-overuse maintained this status after 3 months [28]. One single center, cross-sectional and prospective study from Brazil including *n* = 200 patients with CM and MOH, showed that the mAbs increased the response to an established strategy that included the initiation of a non-CGRP preventive medication [29]. A recent study from Italy included people with MOH receiving anti-CGRP mAbs who underwent in-hospital sudden detoxification and compared them with a sample who did not perform detoxification [30]. There were no differences in response between the two groups, suggesting that anti-CGRP mAbs may be effective in MOH irrespective of detoxification.

### Safety, discontinuation, and dropouts of anti-CGRP mAbs

The adverse event (AE) rate with drugs targeting CGRP was higher in RCTs (30.7–58%) [18–22] than in RWS (7.9–48%) [23–27], with most being mild or moderate. The most common AEs were gastrointestinal symptoms, flu-like symptoms, and injection-site reactions. Serious AEs were rare in all the RCT, with similar rates compared to placebo. There is evidence to suggest that gastrointestinal symptoms are less common with galcanezumab and fremanzeumab than with erenumab [20, 22]. Additionally, discontinuation of a trial due to AEs was infrequent, a finding consistent also in RWS.

One of the most exciting questions regarding CGRP medication is the timing of discontinuation, either in episodic or chronic migraine. Data from RCT and RWS suggests that the effect of anti-CGRP drugs persists for at least up to 3 months after discontinuation [32]. After that, most patients experience worsening of their headaches [32]. A study that included *n* = 44 patients with resistant CM and MOH successfully treated with erenumab or galcanezumab for 12 months, showed that up to 72% worsened after discontinuation (due to government reimbursement practices) [32]. On the other hand, one-quarter of people maintained a clinical response after discontinuation and did not need to restart treatment [32].

### Onabotulinumtoxin-A and anti-CGRP mAbs

*Post hoc* analyses of clinical trials [33] and RWS [25, 27] have shown the efficacy of anti-CGRP mAbs in CM patients non-responsive to BTX-A. One cohort consisted of eighty-two patients who switched from BTX-A to an anti-CGRP mAb after a 6-month interval [35] and found that the mAb was effective in 65% of the population [35]. However, this study did not aim to assess a direct comparison between the two treatments, and excluded super-responders to BTX-A, and as such the results are limited [35].

All RCT excluded concomitant treatment of CGRP drugs and BTX-A, which is an important gap between trial data and real-world challenges. Of note, one study showed that while CM not responding to BTX-A benefited from anti-CGRP mAbs at 3 months of follow-up, dual therapy was not superior to anti-CGRP mAbs in monotherapy [34].

### Gepants

The positive results of atogepant in CM were seen in the pivotal Phase 3 PROGRESS trial evaluating the dose of 60 mg once daily in adults with CM [36]. A total of *n* = 778 PwM were randomized into one of three treatment groups to receive 60 mg QD of atogepant, 30 mg BID of atogepant, or placebo over a 3months. Atogepant significantly decreased MMDs compared to placebo, and led to significant improvements in all secondary endpoints, while showing a safety profile. Relevant exclusion criteria were current diagnosis of new persistent daily headache and failure of > 4 preventive medications.

## Discussion

In this systematic review we explored the evidence of CGRP-targeted medication in chronic migraine with or without medication overuse. Data from both clinical trials and real-life studies show consistent benefits from this drug class. Most available data regard anti-CGRP mAbs; these medications have been shown to decrease headache frequency in CM and are also capable of reverting resistant CM to episodic frequency in a significant portion of patients. Further, the safety profile of these drugs allows for low discontinuation rates [37, 38]. Currently, there is no available data suggesting the superiority of one particular antibody above the others [14, 39]. Gepants, on the other hand, need more data, specifically data from RWS.

The identification of clinical predictors of a good response to anti-CGRP drugs could help personalize the treatment of migraine. Despite some noted conflicting results, there are likely predictive factors of response to anti-CGRP mAbs in CM. While unilateral pain, unilateral autonomical signs, cutaneous allodynia, and lower baseline headache frequency are associated with a better response to anti-CGRP mAbs, obesity seems to be a negative predictive factor of response perhaps due to the association between obesity and higher levels of CGRP. However, evidence is still very scarce, and no definite clinical predictor has still identified. Stopping treatment with this medication in responders may worsen the headaches and, in some cases, may lead back to chronicity [32]. However, re-initiation seems to quickly lead to new improvements, and it is still unclear for how long the medication should be given. This data is not available yet for gepants.

Data from animal models have shown that mAbs are effective in the prevention and treatment of MOH, measured as cutaneous allodynia, even after a single administration [40]. In one study, the nitroglycerin-induced upregulation of trigeminal nucleus caudalis FOS-positive cells was inhibited by pretreatment with olcegepant, suggesting that CGRP may be important in an early phase of nitroglycerin-induced central trigeminal activity [41].

In this systematic review, we also found evidence for the benefit of anti-CGRP drugs in CM with MOH, with significant high rates of conversion from MOH to non-MOH [23–25]. This is seen with medication overuse with simple analgesics, triptans, and combination therapy [28]. The evidence in people with opioid overuse, however, is much lower, as opioid overuse is often an exclusion criterion in clinical trials [18, 20, 22]. Also noteworthy is that the anti-CGRP drugs may be effective regardless of the presence of acute medication overuse [30]. One distinct aspect of gepants is the apparent absence of MOH risk, even when these are used as acute treatment, which is mainly based on preclinical data showing that latent sensitization or cutaneous allodynia are not induced by these drugs [42]. One possible reason is the level at gepants act, which is post-synaptic, as opposed to ditans that are pre-synaptic and increase CGRP expression. The preclinical data agrees with preliminary clinical results, which show no evidence of MOH development after exposure to gepants [42, 43].

We must consider the strengths and limitations of the studies. RCTs are of obvious importance to understand the efficacy and safety of drug class. However, in most clinical trials on CM treated with anti-CGRP mAbs, other migraine preventive drugs were prohibited during the study and up to 2–3 months before starting the baseline. Additionally, several RCTs preclude the inclusion of people with more refractory diseases, by putting a cap on the number of previous preventive failures [19–22]. Despite their various limitations, studies using real-world data have the advantage of involving a broader population. RWS are probably a better representation of people with CM found in clinical practice, who are often very difficult to treat and have complex comorbidities. A potential limitation in RWS, and particularly retrospective ones, is that treatment failure is usually based on medical history and clinical judgment, without the objective cut-offs used in clinical trials. However, while the absence of objective rating scales might limit data interpretation, in clinical practice, we often rely on patients´s subjective reporting.

Although targeting the CGRP pathway is an effective approach in migraine treatment, including CM, a significant portion of patients do not respond to this medication. In fact, up to 15–25% of the patients with migraine treated with an anti-CGRP mAbs are found to discontinue treatment due to lack of efficacy [44]. In this regard, one thing to take into consideration is the timing to assess efficacy, as some cases of CM may present a late response, even after 12 weeks of treatment [45]. The method used to assess efficacy might also influence the continuation. Assessing disease burden might be more sensitive than headache frequency, for example [27]. Also, despite the low evidence, there is likely a rationale for switching among the mAbs, from an anti-CGRP receptor to an anti-CGRP ligand and vice-versa. This was seen in a small retrospective study from Germany that included twenty-five non-responders to erenumab who switched to galcanezumab or fremanzeumab, finding a clinical response in one-third of subjects [44]. Interestingly, none of the patients with daily headache responded to the antibody switch [44]. There are currently no data on switching from anti-CGRP mAbs and gepants.

Possible explanations for the failure of CGRP blocking treatments include individual factors such as CYT genotype, BMI, and lipophilic index [46]. Furthermore, the CGRP pathway is likely not the only pathway involved in migraine attacks. Other molecules include adenosine receptors A1/A2A, glutamate receptors, pituitary adenylate cyclase-activating peptide (PACAP) receptors, delta-opioid receptors (DORS), acid-sensing ion channels (ASICs) and amylin receptors [47].

The experience with the use of anti-CGRP medication has contributed to a better understanding of migraine pathophysiology. Although most of the anti-CGRP action seems to occur outside of the blood-brain barrier (BBB) [48], the clinical data shows that central symptoms of migraine can respond to anti-CGRP drugs, suggesting that effects of CGRP within meningeal trigeminal afferents can counteract the input of key CNS structures involved in the CGRP pathway. Interestingly, in a mouse model of post-traumatic headache, the early administration of fremanezumab following mild traumatic brain injury prevented the development of cutaneous allodynia, as well as the loss of net descending pain inhibitory control pathway, suggesting that these drugs can affect the migraine matrix [49].

Targeting different pathways involved in migraine physiology has been the basis of combining different medications. In this regard, adding an anti-CGRP mAb to BTX-A in CM has been considered a promising dual therapy. While some experts consider that there is not enough evidence to combine BTX-A and anti-CGRP mAbs [50], others suggest that this dual targeting therapy might be beneficial to difficult-to-treat cases of CM [51]. Indeed, while BTX-A acts peripherally inhibiting the release of pain-modulating substances, including CGRP, from extracranial and meningeal C-fibers, the anti-CGRP mAbs act more systemically on CGRP ligand and receptor interaction, predominantly within meningeal vessel walls and meningeal Aδ-fibers [52, 53]. However, most clinical trials with anti-CGRP medication excluded the concomitant use of BTX-A [18–22]. Thus, the available data on the combination of anti-CGRP and BTX-A comes from the real world only and suggests promise in difficult to treat cases [54, 55]. One small retrospective multicenter study assessed the effectiveness of combining dual therapy with BTX-A add-on to anti-CGRP mAb (erenumab or fremanezumab) in treatment-refractory CM who failed to respond to adequate monotherapy with three courses of BTX-A [56]. Of note, previously switching from BTX-A to anti-CGRP mAb monotherapy had not been effective. Despite the small sample (*n* = 19), the authors found a response of 74% to the combined treatment [56]. Interestingly, neck pain was associated with a greater response to dual therapy [56]. Another small case series (*n* = 17) showed a good response to dual therapy in people with partial or no response to BTX-A [57].

## Conclusions

The anti-CGRP drugs offer an effective and well-tolerated option for migraine treatment, particularly in chronic migraine. However, several questions remain on the use of these drugs, including the benefit of combined treatment with other migraine preventives and switching to other drugs of the same class.

### Electronic supplementary material

Below is the link to the electronic supplementary material.

Supplementary Material 1

## Data Availability

NA (review article).

## References

[1] May A, Schulte LH (2016). Chronic migraine: risk factors, mechanisms and treatment. Nat Rev Neurol.

[2] Burch RC, Buse DC, Lipton RB, Migraine (2019). Epidemiology, Burden, and Comorbidity. Neurol Clin.

[3] Vandenbussche N, Laterza D, Lisicki M, Lloyd J, Lupi C, Tischler H (2018). Medication-overuse headache: a widely recognized entity amidst ongoing debate. J Headache Pain.

[4] Buse DC, Reed ML, Fanning KM, Bostic R, Dodick DW, Schwedt TJ (2020). Comorbid and co-occurring conditions in migraine and associated risk of increasing headache pain intensity and headache frequency: results of the migraine in America symptoms and treatment (MAST) study. J Headache Pain.

[5] Diener HC, Holle D, Solbach K, Gaul C (2016). Medication-overuse headache: risk factors, pathophysiology and management. Nat Rev Neurol.

[6] Al Ghadeer HA, AlSalman SA, Albaqshi FM, Alsuliman SR, Alsowailem FA, Albusror HA (2021). Quality of life and disability among Migraine patients: a single-center study in AlAhsa, Saudi Arabia. Cureus.

[7] Buse DC, Scher AI, Dodick DW, Reed ML, Fanning KM, Manack Adams A et al (2016) Impact of Migraine on the Family: Perspectives of People With Migraine and Their Spouse/Domestic Partner in the CaMEO Study. Mayo Clin Proc. ;S0025-6196(16)00126-9 10.1016/j.mayocp.2016.02.013 27132088

[8] Schytz HW, Amin FM, Jensen RH, Carlsen L, Maarbjerg S, Lund N et al (2020) Reference programme: diagnosis and treatment of headache disorders and facial pain. Danish Headache Society, 3rd edition, J Headache Pain. 2021;22(1):22 10.1186/s10194-021-01228-4 PMC8034101 33832438

[9] Agostoni EC, Barbanti P, Calabresi P, Colombo B, Cortelli P, Frediani F (2019). Current and emerging evidence-based treatment options in chronic migraine: a narrative review. J Headache Pain.

[10] Hepp Z, Dodick DW, Varon SF, Gillard P, Hansen RN, Devine EB (2015). Adherence to oral migraine-preventive medications among patients with chronic migraine. Cephalalgia Int J Headache.

[11] Berger A, Bloudek LM, Varon SF, Oster G (2012). Adherence with migraine prophylaxis in clinical practice. Pain Pract off J World Inst Pain.

[12] Silberstein SD, Dodick DW, Aurora SK, Diener HC, DeGryse RE, Lipton RB (2015). Per cent of patients with chronic migraine who responded per onabotulinumtoxinA treatment cycle: PREEMPT. J Neurol Neurosurg Psychiatry.

[13] Blumenfeld AM, Frishberg BM, Schim JD, Iannone A, Schneider G, Yedigarova L (2021). Real-world evidence for control of chronic migraine patients receiving CGRP monoclonal antibody Therapy added to OnabotulinumtoxinA: a Retrospective Chart Review. Pain Ther.

[14] T S, K B, A T. Calcitonin gene relating peptide inhibitors in combination for migraine treatment: A mini-review. Front Pain Res Lausanne Switz [Internet]. (2023) Mar 17 [cited 2024 Jan 7];4. Available from: https://pubmed.ncbi.nlm.nih.gov/37006413/10.3389/fpain.2023.1130239 PMC10064089 37006413

[15] Alpuente A, Torres-Ferrus M, Terwindt GM (2023). Preventive CGRP-targeted therapies for chronic migraine with and without medication-overuse headache. Cephalalgia.

[16] Sacco S, Amin FM, Ashina M, Bendtsen L, Deligianni CI, Gil-Gouveia R (2022). European Headache Federation guideline on the use of monoclonal antibodies targeting the calcitonin gene related peptide pathway for migraine prevention – 2022 update. J Headache Pain.

[17] Moher D, Liberati A, Tetzlaff J, Altman DG, PRISMA Group (2010). Preferred reporting items for systematic reviews and meta-analyses: the PRISMA statement. Int J Surg Lond Engl.

[18] Lipton RB, Goadsby PJ, Smith J, Schaeffler BA, Biondi DM, Hirman J et al Efficacy and safety of eptinezumab in patients with chronic migraine: PROMISE-2. Neurology [Internet]. 2020 Mar 31 [cited 2024 Jan 7];94(13). Available from: https://www.neurology.org/doi/10.1212/WNL.0000000000009169 10.1212/WNL.0000000000009169 PMC7274916 32209650

[19] Sakai F, Suzuki N, Kim BK, Igarashi H, Hirata K, Takeshima T (2021). Efficacy and safety of fremanezumab for chronic migraine prevention: Multicenter, randomized, double-blind, placebo-controlled, parallel-group trial in Japanese and Korean patients. Headache.

[20] Detke HC, Goadsby PJ, Wang S, Friedman DI, Selzler KJ, Aurora SK (2018). Galcanezumab in chronic migraine: the randomized, double-blind, placebo-controlled REGAIN study. Neurology.

[21] Tepper S, Ashina M, Reuter U, Brandes JL, Doležil D, Silberstein S (2017). Safety and efficacy of erenumab for preventive treatment of chronic migraine: a randomised, double-blind, placebo-controlled phase 2 trial. Lancet Neurol.

[22] Silberstein SD, Dodick DW, Bigal ME, Yeung PP, Goadsby PJ, Blankenbiller T (2017). Fremanezumab for the Preventive treatment of chronic migraine. N Engl J Med.

[23] Pensato U, Baraldi C, Favoni V, Cainazzo MM, Torelli P, Querzani P (2022). Real-life assessment of erenumab in refractory chronic migraine with medication overuse headache. Neurol Sci off J Ital Neurol Soc Ital Soc Clin Neurophysiol.

[24] Curone M, Tullo V, Didier HA, Bussone G (2022). Overview on effectiveness of erenumab, fremanezumab, and galcanezumab in reducing medication overuse headache in chronic migraine patients. Neurol Sci off J Ital Neurol Soc Ital Soc Clin Neurophysiol.

[25] Russo A, Silvestro M, Scotto di Clemente F, Trojsi F, Bisecco A, Bonavita S (2020). Multidimensional assessment of the effects of erenumab in chronic migraine patients with previous unsuccessful preventive treatments: a comprehensive real-world experience. J Headache Pain.

[26] Lambru G, Hill B, Murphy M, Tylova I, Andreou AP (2020). A prospective real-world analysis of erenumab in refractory chronic migraine. J Headache Pain.

[27] Iannone LF, Fattori D, Benemei S, Chiarugi A, Geppetti P, De Cesaris F (2022). Long-term effectiveness of three Anti-CGRP monoclonal antibodies in resistant chronic migraine patients based on the MIDAS score. CNS Drugs.

[28] Tepper SJ, Diener HC, Ashina M, Brandes JL, Friedman DI, Reuter U (2019). Erenumab in chronic migraine with medication overuse: subgroup analysis of a randomized trial. Neurology.

[29] Krymchantowski AV, Jevoux C, Krymchantowski AG, Silva-Néto RP (2023). Monoclonal antibodies for chronic migraine and medication overuse headache: a real-world study. Front Neurol.

[30] Pensato U, Baraldi C, Favoni V, Mascarella D, Matteo E, Andrini G (2022). Detoxification vs non-detoxification before starting an anti-CGRP monoclonal antibody in medication overuse headache. Cephalalgia Int J Headache.

[31] Barbanti P, Egeo G, Aurilia C, Altamura C, d’Onofrio F, Finocchi C (2022). Predictors of response to anti-CGRP monoclonal antibodies: a 24-week, multicenter, prospective study on 864 migraine patients. J Headache Pain.

[32] Iannone LF, Fattori D, Benemei S, Chiarugi A, Geppetti P, De Cesaris F (2022). Predictors of sustained response and effects of the discontinuation of anti-calcitonin gene related peptide antibodies and reinitiation in resistant chronic migraine. Eur J Neurol.

[33] Ailani J, Pearlman E, Zhang Q, Nagy AJ, Schuh K, Aurora SK (2020). Positive response to galcanezumab following treatment failure to onabotulinumtoxinA in patients with migraine: post hoc analyses of three randomized double-blind studies. Eur J Neurol.

[34] Alpuente A, Gallardo VJ, Caronna E, Torres-Ferrús M, Pozo-Rosich P (2021). Partial and nonresponders to onabotulinumtoxinA can benefit from anti-CGRP monoclonal antibodies preventive treatment: a real-world evidence study. Eur J Neurol.

[35] Iannone LF, Fattori D, Marangoni M, Benemei S, Chiarugi A, Geppetti P (2023). Switching OnabotulinumtoxinA to monoclonal Anti-CGRP antibodies in drug-resistant chronic migraine. CNS Drugs.

[36] Pozo-Rosich P, Ailani J, Ashina M, Goadsby PJ, Lipton RB, Reuter U (2023). Atogepant for the preventive treatment of chronic migraine (PROGRESS): a randomised, double-blind, placebo-controlled, phase 3 trial. Lancet Lond Engl.

[37] Ornello R, Casalena A, Frattale I, Gabriele A, Affaitati G, Giamberardino MA (2020). Real-life data on the efficacy and safety of erenumab in the Abruzzo region, central Italy. J Headache Pain.

[38] Messina R, Huessler EM, Puledda F, Haghdoost F, Lebedeva ER, Diener HC (2023). Safety and tolerability of monoclonal antibodies targeting the CGRP pathway and gepants in migraine prevention: a systematic review and network meta-analysis. Cephalalgia Int J Headache.

[39] Haghdoost F, Puledda F, Garcia-Azorin D, Huessler EM, Messina R, Pozo-Rosich P (2023). Evaluating the efficacy of CGRP mAbs and gepants for the preventive treatment of migraine: a systematic review and network meta-analysis of phase 3 randomised controlled trials. Cephalalgia Int J Headache.

[40] Kopruszinski CM, Xie JY, Eyde NM, Remeniuk B, Walter S, Stratton J (2017). Prevention of stress- or nitric oxide donor-induced medication overuse headache by a calcitonin gene-related peptide antibody in rodents. Cephalalgia Int J Headache.

[41] Feistel S, Albrecht S, Messlinger K (2013). The calcitonin gene-related peptide receptor antagonist MK-8825 decreases spinal trigeminal activity during nitroglycerin infusion. J Headache Pain.

[42] Saengjaroentham C, Strother LC, Dripps I, Sultan Jabir MR, Pradhan A, Goadsby PJ (2020). Differential medication overuse risk of novel anti-migraine therapeutics. Brain J Neurol.

[43] Puledda F, Silva EM, Suwanlaong K, Goadsby PJ (2023). Migraine: from pathophysiology to treatment. J Neurol.

[44] Overeem LH, Peikert A, Hofacker MD, Kamm K, Ruscheweyh R, Gendolla A (2022). Effect of antibody switch in non-responders to a CGRP receptor antibody treatment in migraine: a multi-center retrospective cohort study. Cephalalgia Int J Headache.

[45] Barbanti P, Aurilia C, Egeo G, Torelli P, Proietti S, Cevoli S (2023). Late response to Anti-CGRP monoclonal antibodies in migraine: a Multicenter prospective observational study. Neurology.

[46] Al-Hassany L, Goadsby PJ, Danser AHJ, MaassenVanDenBrink A (2022). Calcitonin gene-related peptide-targeting drugs for migraine: how pharmacology might inform treatment decisions. Lancet Neurol.

[47] Dussor G (2019). New discoveries in migraine mechanisms and therapeutic targets. Curr Opin Physiol.

[48] Johnson KW, Morin SM, Wroblewski VJ, Johnson MP (2019). Peripheral and central nervous system distribution of the CGRP neutralizing antibody [125I] galcanezumab in male rats. Cephalalgia Int J Headache.

[49] Kopruszinski CM, Turnes JM, Swiokla J, Weinstein TJ, Schwedt TJ, Dodick DW (2021). CGRP monoclonal antibody prevents the loss of diffuse noxious inhibitory controls (DNIC) in a mouse model of post-traumatic headache. Cephalalgia Int J Headache.

[50] Sacco S, Braschinsky M, Ducros A, Lampl C, Little P, van den Brink AM (2020). European headache federation consensus on the definition of resistant and refractory migraine: developed with the endorsement of the European Migraine & Headache Alliance (EMHA). J Headache Pain.

[51] Ailani J, Burch RC, Robbins MS, Board of Directors of the American Headache Society (2021). The American Headache Society Consensus Statement: update on integrating new migraine treatments into clinical practice. Headache.

[52] Burstein R, Blumenfeld AM, Silberstein SD, Manack Adams A, Brin MF (2020). Mechanism of action of OnabotulinumtoxinA in chronic migraine: a narrative review. Headache.

[53] Melo-Carrillo A, Strassman AM, Schain AJ, Noseda R, Ashina S, Adams A (2019). Exploring the effects of extracranial injections of botulinum toxin type A on prolonged intracranial meningeal nociceptors responses to cortical spreading depression in female rats. Cephalalgia Int J Headache.

[54] Ferrari MD, Zuurbier KWM, Barash S, Ning X, Cohen JM (2022). Fremanezumab in individuals with chronic migraine who had inadequate response to onabotulinumtoxinA and topiramate or valproic acid. Headache.

[55] Ashina M, Tepper S, Brandes JL, Reuter U, Boudreau G, Dolezil D (2018). Efficacy and safety of erenumab (AMG334) in chronic migraine patients with prior preventive treatment failure: a subgroup analysis of a randomized, double-blind, placebo-controlled study. Cephalalgia Int J Headache.

[56] Argyriou AA, Dermitzakis EV, Xiromerisiou G, Vikelis M (2022). OnabotulinumtoxinA Add-On to monoclonal Anti-CGRP antibodies in treatment-refractory chronic migraine. Toxins.

[57] Toni T, Tamanaha R, Newman B, Liang Y, Lee J, Carrazana E (2021). Effectiveness of dual migraine therapy with CGRP inhibitors and onabotulinumtoxinA injections: case series. Neurol Sci off J Ital Neurol Soc Ital Soc Clin Neurophysiol.

