# Supplementary material for: CGRP-targeted medication in chronic migraine - systematic review
Source: J Headache Pain. 2024 Apr 5;25(1):51. doi: 10.1186/s10194-024-01753-y (PMC10996229; doi:10.1186/s10194-024-01753-y)
Supplement: Supplementary file 1 — Supplementary Material 1 [file 10194_2024_1753_MOESM1_ESM.docx]

**CGRP-TARGETED MEDICATION IN CHRONIC MIGRAINE**

Renato Oliveira, Raquel Gil-Gouveia, Francesca Puledda

**SUPPLEMENTAL MATERIALS**

**SUPPLEMENTAL METHODS**

**Search algorithm used in MEDLINE search**

| # | SEARCHES | RESULTS |
| --- | --- | --- |
| 1 | migraine[MeSH Terms] | 47006 |
| 2 | headache[MeSH Terms] | 118237 |
| 3 | (((migraine or migraines or “chronic migraine”) or ((headache or headaches))) OR ((“migraine attack”)) OR (“head pain” or “trigeminal pain”))) | 113123 |
| 4 | 1 or 2 or 3 | 139134 |
| 5 | CGRP | 11289 |
| 6 | Anti-CGRP | 288 |
| 7 | ((“CGRP medication” or “CGRP drugs” or “CGRP antagonist")) | 1090 |
| 8 | 6 or 7 | 1306 |
| 9 | 5 and 8 | 1306 |
| 10 | erenumab or fremanezumab or galcanezumab or eptinezumab | 858 |
| 11 | Gepant* or atogepant or rimegepant | 310 |
| 12 | 10 or 11 | 1108 |
| 13 | 4 and 9 and 12 | 275 |

**Figure 1 – Flowchart summarizing literature search strategy**

**
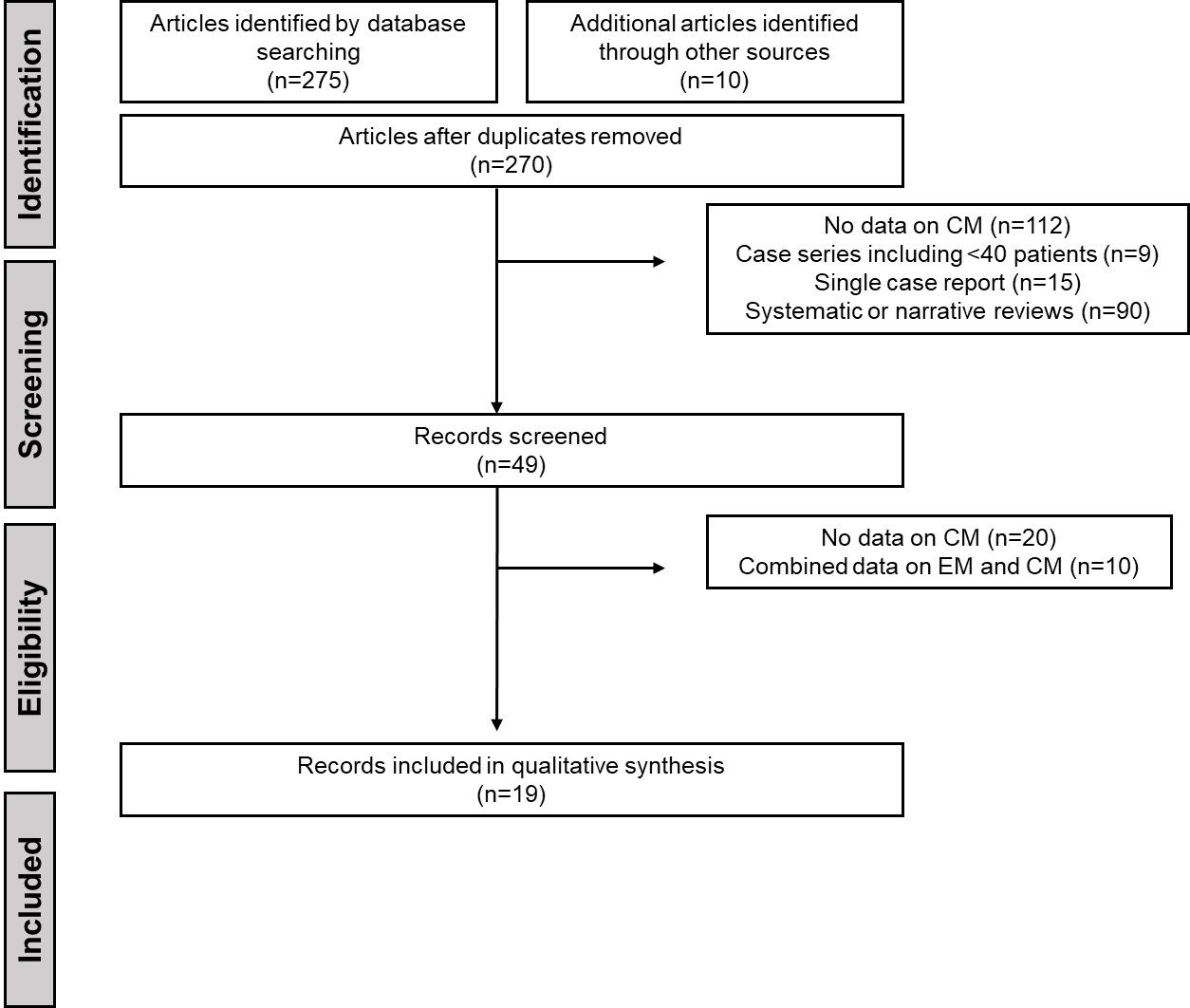
**

**Supplemental Table I – Summary of Quality analysis of the included studies using the Newcastle-Ottawa Quality Assessment Scale for cohort studies**

| **Author, year** | **Selection** | | | | **Comparability** | **Outcome and reporting** | | | **Score** | **Overall quality** |
| --- | --- | --- | --- | --- | --- | --- | --- | --- | --- | --- |
|  | **Representativeness** | **Selection of the cohort** | **Ascertainment of exposure** | **Baseline assessment** |  | **Assessment of outcome** | **Follow-up length** | **Adequacy of ascertainment** |  |  |
| **Richard B. Lipton et al., 2020** | 0 | 1 | 1 | 1 | 2 | 1 | 0 | 1 | 7 | Moderate |
| **Fumihiko Sakai, et al., 2021** | 0 | 1 | 1 | 1 | 2 | 1 | 0 | 1 | 7 | Moderate |
| **Holland C Detke, et al., 2018** | 0 | 1 | 1 | 1 | 2 | 1 | 1 | 1 | 8 | High |
| **Stephen D Silberstein, et al., 2017** | 1 | 1 | 1 | 1 | 2 | 1 | 0 | 1 | 8 | High |
| **Stewart J Tepper, et al., 2017** | 0 | 1 | 1 | 1 | 2 | 1 | 1 | 1 | 8 | High |
| **Luigi F. Iannone et al., 2022** | 1 | 1 | 1 | 1 | 1 | 1 | 1 | 1 | 8 | High |
| **Umberto Pensato et al., 2022** | 1 | 1 | 1 | 1 | 1 | 1 | 0 | 1 | 7 | Moderate |
| **Marcella Curone et al., 2022** | 0 | 1 | 1 | 1 | 1 | 1 | 1 | 1 | 7 | Moderate |
| **Giorgio Lambru et al., 2020** | 1 | 1 | 1 | 1 | 1 | 1 | 1 | 1 | 8 | High |
| **Antonio Russo et al., 2020** | 1 | 1 | 1 | 1 | 1 | 1 | 1 | 1 | 8 | High |
| **Stewart J Tepper et al., 2019** | 0 | 1 | 1 | 1 | 2 | 1 | 0 | 1 | 7 | Moderate |
| **Abouch V. Krymchantowski, et al., 2023** | 0 | 1 | 1 | 1 | 1 | 1 | 0 | 1 | 6 | Moderate |
| **Umberto Pensato, et al., 2022** | 0 | 1 | 1 | 1 | 2 | 1 | 1 | 1 | 8 | High |
| **Luigi Francesco Iannone, et al., 2022** | 0 | 1 | 1 | 1 | 1 | 1 | 0 | 1 | 6 | Moderate |
| **Jessica Ailani, et al., 2020** | 0 | 1 | 1 | 1 | 2 | 1 | 0 | 1 | 7 | Moderate |
| **Alicia Alpuente, et al., 2021** | 0 | 1 | 1 | 1 | 1 | 1 | 0 | 1 | 6 | Moderate |
| **Patricia Pozo-Rosic, et al., 2023** | 1 | 1 | 1 | 1 | 2 | 1 | 0 | 1 | 8 | High |
| **Piero Barbanti, et al., 2022** | 1 | 1 | 1 | 1 | 1 | 1 | 1 | 1 | 8 | High |
| **Luigi F. Iannone, et al., 2023** | 0 | 1 | 1 | 1 | 1 | 1 | 1 | 1 | 7 | Moderate |
